# Nano-Chemotherapy synergize with immune checkpoint inhibitor- A better option?

**DOI:** 10.3389/fimmu.2022.963533

**Published:** 2022-08-09

**Authors:** Xinye Qian, Wang Hu, Jun Yan

**Affiliations:** ^1^ Center of Hepatobiliary Pancreatic Disease, Beijing Tsinghua Changgung Hospital, School of Clinical Medicine, Tsinghua University, Beijing, China; ^2^ School of Clinical Medicine, Tsinghua University, Beijing, China

**Keywords:** nano-chemotherapy, immune checkpoint inhibitor, treatment efficiency, tumor, toxicity

## Abstract

Immune checkpoint inhibitor (ICI) is one of the most important tumor treatment methods. Although the therapeutic efficiency of immune checkpoint inhibitor mono-therapy is limited, the combination of chemotherapy plus immune checkpoint inhibitors has shown great advantages in cancer treatment. This is mainly due to the fact that tumor reactive T cells could fully provide their anti-tumor function as chemotherapy could not only cause immunogenic cell death to increase antigen presentation, but also improve the immunosuppressive tumor micro-environment to synergize with immune checkpoint inhibitors. However, traditional chemotherapy still has shortcomings such as insufficient drug concentration in tumor region, short drug duration, drug resistance, major adverse events, etc, which might lead to the failure of the therapy. Nano chemotherapeutic drugs, which refer to chemotherapeutic drugs loaded in nano-based drug delivery system, could overcome the above shortcomings of traditional chemotherapeutic drugs to further improve the therapeutic effect of immune checkpoint inhibitors on tumors. Therefore, the scheme of nano chemotherapeutic drugs combined with immune checkpoint inhibitors might lead to improved outcome of cancer patients compared with the scheme of traditional chemotherapy combined with immune checkpoint inhibitors.

## Introduction

The development of immune checkpoint inhibitors (ICI) has brought new opportunities for tumor patients (1). Mainly two kinds of immune checkpoint inhibitors are currently applied in clinic, including CTLA-4 inhibitors (2) and PD-1/PD-L1 inhibitors (3). Moreover, novel immune checkpoint inhibitors, like TIGIT inhibitor (4), are under clinical trials. Its main principle is to restore the anti-tumor function of tumor-reactive T cells by blocking immune regulation (inhibition) pathways (5). However, the theoretically perfect immune checkpoint inhibitor mono-therapy has not exceeded traditional cancer treatment in many tumors like pancreatic cancer, breast cancer, etc (6). To Improve treatment effect, the combination of immune checkpoint inhibitors and chemotherapy has been invented. The combination has already shown improved therapeutic effects on a number of tumors, including esophageal cancer, lung cancer, triple-negative breast cancer and so on (7). In this paper, nano chemotherapeutic drugs are suggested as a better option for combined immune checkpoint inhibitor therapy to achieve better therapeutic effect in tumor patients by analyzing the therapeutic principle behind this combination of immune checkpoint inhibitor plus chemotherapy, and by comparing the differences between Nano chemotherapeutic drugs and traditional chemotherapeutic drugs.

## Mechanism to improve the therapeutic effect of immune checkpoint inhibitor

The rationale of immune checkpoint inhibitor therapy is based on the anti-tumor function of tumor reactive T cells (CD8+ cytotoxic T lymphocytes) (8), which depend not only on the inhibition of immunosuppressive pathways, but also on other mechanisms.

First, the immune system must recognize tumor cells, which depends on presentation of tumour antigens by antigen-presenting cells (APC) (9). Apoptosis of tumor cells with new antigens might not start the antigen presentation process (10), which would lead to the failure of the immune system recognizing tumor cells. Only when immunogenic cell death (ICD, a type of cancer cell death involves the activation of the immune system against cancer in immunocompetent hosts) were triggered, APC cells would be activated and present the specific antigens of tumor cells to tumor reactive T cells so that these T cells could recognize tumor cells and produce anti-tumor effect (11).

Second, tumor reactive T cells that could recognize tumor antigens need to contact with tumor cells to provide their anti-tumor effect. However, tumors develop multiple mechanisms to escape the “hunt” from tumor reactive T cells. One way is to exhaust tumor reactive T cells to impair their anti-tumor function, like expressing immune checkpoint molecules (such as PD-L1) to induce tumor reactive T cells apoptosis or exhaustion by activating immunosuppressive pathways (12), or recruiting immunosuppressive cells such as tumor associated macrophages (TAM), tumor associated fibroblasts (CAF), regulatory T cells (Tregs) and myelogenous suppressor cells (MDSCs) to suppress the activity of cytotoxic T lymphocytes (CTLs) (13, 14).

Another way is to reduce the number of tumor reactive T cells at the tumor site. The failure to attract tumour-reactive T cells to the tumour could be caused by the lack of appropriate chemokine secretion from the tumor (e.g. the down regulation of CXCL9 prevents CD8+ T cell tumor-infiltration. Thus, impairing anti-PD1 therapy) (15). The activity of immunosuppressive cells is also important for suppressing the infiltration of CTLs into the tumor area; for example, CAF could release chemokine CXCL12, which could inhibit T cell infiltration in tumors (16). Also, this immunosuppressive micro-environment could reduce the recruitment of DC cells so that tumor antigens cannot be presented, resulting in the failure of the adaptive immune system to recognize tumor cells (16). Moreover, CTLs are not generally able to reach the edge area of some tumors due to trapping within the stroma of tumor or in the peri-tumoral tissue because of a unique architecture of tumor immunosuppressive environment (16).

Therefore, if tumor antigen presentation and tumor reactive T cells infiltration could be ensured, the therapeutic efficiency of immune checkpoint inhibitors on tumors could be improved as tumor reactive T cells could fully act its anti-tumor effect.

## Mechanism and therapeutic effect of chemotherapy plus immune checkpoint inhibitor

Immune checkpoint inhibitor combined with chemotherapy has been proved to increase the efficiency of tumor treatment in a variety of clinical studies. For example, nivolumab (PD-1 antibody) plus first-line chemotherapy resulted in significantly longer overall survival than chemotherapy alone in patients with advanced esophageal squamous-cell carcinomaincluding esophageal cancer (13.2 months vs 10.7 month) (17); the combination of pembrolizumab (PD-1 antibody) with standard chemotherapy of pemetrexed and a platinum-based drug resulted in significantly longer overall survival and progression-free survival than chemotherapy alone in metastatic non-small cell Lung Cancer (18); Pembrolizumab plus chemotherapy showed a significant and clinically meaningful improvement in progression-free survival versus placebo-chemotherapy among patients with metastatic triple-negative breast cancer with CPS (combined positive score) of 10 or more (19); etc. The underlying mechanism is that while immune checkpoint inhibitors block the immunosuppressive pathways, chemotherapy might improve the presentation of tumor antigen and the infiltration of tumor reactive T cells.

On the one hand, traditional chemotherapy could lead to immunogenic cell death (ICD) of tumor cell (20), which could promote the presentation of tumor antigens so that the adaptive immune system could recognize tumor cells. Importantly, the molecular mechanism by which chemotherapeutic drugs activate the immune activation pathway does not necessarily overlap with its cytotoxic mechanism. It is reported that the DNA-intercalating agents adriamycin and oxaliplatin, mainly inhibit topoisomerase II at clinically relevant doses, could also induce eIF2α phosphorylation in enucleated cancer cells. This means that these chemotherapeutic drugs could act on cytoplasmic (extra-nuclear) structures to stimulate ICD related stress pathways (21), suggesting that the traditional chemotherapy drugs could still promote antigen presentation of tumor cells even if they fail to cause tumor cell death directly. Recent studies found that a variety of chemotherapy drugs, including carboplatin (22) and gemcitabine (23), could promote the recruitment of DC cells in tumor micro-environment, further indicating that they could facilitate the adaptive immune system to recognize tumor cells. The mechanism might be as follows: the activation of ICD-linked danger signaling; the elevation of cytokine secretion, such as type I IFNs; the reversal of immunosuppressive micro-environment, such as the depletion of TAMs, the decreased secretion of TGFβ, etc.

**Figure 1:**
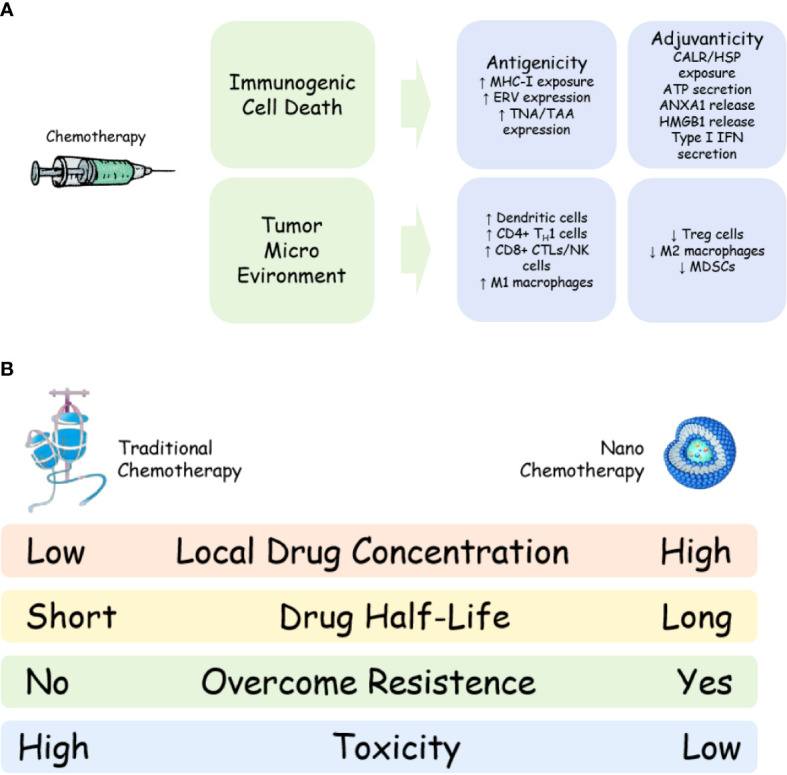
Mechanism and advantage of nano-chemotherapy. **(A)** Mechanism of chemotherapy to synergize with immune checkpoint inhibitor; **(B)** Advantages of nano-chemotherapy compared with traditional chemotherapy. MHC-I, major histocompatibility complex class I;ERV, endogenous retrovirus; TAA, tumour associated antigen; TNA, tumour neoantigen; CALR, calreticulin; HSP, heat shock protein;ANXA1, annexin A1; HMGB1, high mobility group box 1; IFN, interferon; TH1, T helper 1; CTL, cytotoxic T lymphocyte; NK, natural killer cells; MDSC, myeloid-derived suppressor cell; Treg, regulatory T cells.

On the other hand, traditional chemotherapy could reverse the immunosuppressive tumor micro-environment, including the depletion of immunosuppressive cells, like CD4+CD25+FOXP3+ regulatory T (Treg) cells, myelogenous suppressor cells (MDSCs) and M2 like tumor associated macrophages (TAMs) (24, 25), and the activation of immune effector cells, including M1 like TAMs (26), DCs (27) and CTLs (28). Meanwhile, chemotherapy could also increase reactive T cell infiltration in tumor areas, which further ensures the anti-tumor effect of tumor reactive T cells. A systematic review including 110 studies confirmed that chemotherapy could regulate the tumor immune microenvironment, including increasing infiltration of CD8+ cytotoxic T cells, reduction of FOXP3+ Treg and higher PD-L1 expression (29), proving that traditional chemotherapy could cooperate with immune checkpoint inhibitors to improve the anti-tumor ability of tumor reactive T cells by altering tumor immune micro-environment (**Figure 1A**). The mechanism of increasing infiltration of CD8+ cytotoxic T cells after chemotherapy might be that chemotherapy like gemcitabine, 5-Fluorouracil, etc, might decrease Treg and MDSC numbers and increase pro-inflammatory cytokines (such as IFN-γ, IL-2, IL-6, etc) in the tumor region, leading to T cell infiltration to the tumor (24, 25), though further validation is needed.

Based on the above analysis, the combination of chemotherapy and immune checkpoint inhibitors could be called a “Golden Combination” as tumor reactive T cells to fully exert their anti-tumor effects. This is because chemotherapy could promote tumor antigen presentation and tumor reactive T cells infiltration while the immune checkpoint inhibitor could block the immunosuppressive pathway.

However, the disadvantages of traditional chemotherapy might affect this “Golden Combination”. First of all, traditional chemotherapy is usually administrated by a single dose at a regular interval (the interval of chemotherapy is usually over 3 weeks because of its side effects on patients). Drug concentration at tumor region would rapid decrease after chemotherapy (within 3 days after chemotherapy), which might reduce the tumor’s response to chemotherapy (30). Such a drug delivery method might also produce a drug resistance mechanism in tumor cells by over-expression of membrane transporters, like P-glycoproteins on the membrane surface that could expel chemotherapeutic drugs from cells, causing a decrease in the total amount of drugs in cells and failure of chemotherapy (31). These would result in the failure of combination therapy (immune checkpoint inhibitor plus traditional chemotherapy) because tumor antigen could not be present and tumor immune micro-environment could not be improved.

Second, traditional chemotherapy often causes serious side effects in cancer patients (32). Traditional chemotherapy might suppress the immune system by causing lymphocyte depletion (33, 34), which might be detrimental to cancer treatment basing on normal quantity and function of T cells, such as immune checkpoint inhibitor. Although the depletion of lymphocytes might restart the immune system so that the immune system can better fight against tumors (35), this theory has not been confirmed. In addition, doxorubicin and gemcitabine have been found to increase circulating MDSC cells in patients (36), which might worsen the tumor immune micro-environment. Moreover, traditional chemotherapy would cause multiple side effects, including gastrointestinal toxicity (loss of appetite, nausea, vomiting, diarrhea, malabsorption and disorders), myelosuppression, liver and kidney function damage, etc (37). Immune checkpoint inhibitor would also cause side effects, including cardiotoxicity, abnormal liver function, kidney failure, etc (38). Side effects from both chemotherapy and immune checkpoint inhibitor would decrease patient’s tolerability, leading to increased drop out rate or prolonged treatment interval among cancer patients receiving this combination therapy. As a result, these patients might not get better treatment results. In addition, many chemotherapeutic drugs, such as paclitaxel (39), have to be administrated with steroids to reduce their side effects because of their strong toxic effects; but steroids have immunosuppressive effect in mechanism, which would harm the anti-tumor function of T cells (40).

## Nano chemotherapy

Nano chemotherapeutic drugs, which refer to chemotherapeutic drugs loaded in nano-based drug delivery system, are developed to overcome the shortcomings of traditional chemotherapy, including low bio-availability, low local concentration, short duration, major systemic side effects, etc. There are mainly two kinds of nano chemotherapeutic drugs in clinic currently, including Nab-paclitaxel and liposomal chemotherapeutic drugs (**Table 1**).

**Table 1:** Nano-chemotherapeutic Drugs approved in clinic.

Product name	Drug	Targeted tumor	Main cytokines	Immune infiltrate	Reference
Doxil/Caelyx	Liposomal Doxorubicin	Breast cancer; Kaposi’s sarcoma; Ovarian cancer	↑IL-1β, IL-12, IFNγ	↑DCs, CD8+ CTLs, CD4+ T cells; ↓MDSCs, Treg cells	(41)
Myocet	Liposomal Doxorubicin	Breast cancer			(42)
DaunoXome	Liposomal Daunorubicin	Kaposi’s sarcoma	↑Type I IFNs, IFNγ, IL-17	↑DCs, CD8+ CTLs, NK cells; ↓Treg cells	(43)
Lipusu	Liposomal Paclitaxel	Gastric cancer	↑IL-1β, IL-12, TNF	↑DCs, M1 macrophages; ↓Treg cells	(44)
Abraxane	Nab-paclitaxel	Breast Cancer; pancreatic cancer; Non-small cell lung cancer			(45)
Endo-Tag-1	Cationic liposomal paclitaxel	Solid tumors			(46)
Marqibo	LiposomalVincristine	Solid Tumors; Acute lymphoblastic leukemia	ND	↑DCs	(47)
Onivyde	LiposomalIrinotecan	Pancreatic cancer	ND	↑DCs, CD8+ CTLs	(48)
CPX-1	LiposomalIrinotecan	Colorectal cancer			(49)
SPI-077	Liposomal Cis-platin	Solid tumors	↑Type I IFNs, IFNγ	↑DCs, CD8+ CTLs, NK cells	(50)
Lipoplatin	Liposomal Cis-platin	Ovarian cancer; Metastatic non-small cell lung cancer; breast cancer			(51)

IL, interleukin; IFN, interferon; CTL, cytotoxic T lymphocyte; DC, dendritic cell; MDSC, myeloid-derived suppressor cell; NK, natural killer; NKT, natural killer T; ND, not determined; Treg, regulatory T cell.

**Table 1** Nano-chemotherapeutic Drugs approved in clinic.

Nab-paclitaxel is a 130 nm particle formulation comprising albumin nanoparticles and paclitaxel with non-covalent bonds, which could largely reduce the adverse effect of solvent-based paclitaxel, including bone marrow suppression, allergic reactions, neurotoxicity and systemic toxicity (52). Food and Drug Administration (FDA) lists nab-paclitaxel as a vital drug for the treatment of non-small cell lung cancers, pancreatic cancers and breast cancers. Patients with relapsed small cell lung cancer who received nab-paclitaxel had a response rate of 29.4%, prolonged progression-free survival (48 days), and prolonged overall survival (134 days) (53). A systematic review including 63 studies has shown that nab-Paclitaxel continues to demonstrate promising efficacy in breast cancer, including high pathological complete response rates in early-stage breast cancer, particularly in triple-negative breast cancer, and encouraging overall survival in metastatic breast cancer across doses and schedules (54). Furthermore, nab-paclitaxel plus gemcitabine significantly improved overall survival, progression-free survival, and response rate in patients with advanced pancreatic cancer with acceptable adverse events (55), suggesting nab-paclitaxel could combine with other chemotherapy or anti-tumor therapy.

Liposomal chemotherapeutic drugs are chemotherapeutic drugs loaded in liposomes (a revolutionizing nano carrier). Their success is attributed to stable drug loading, extended pharmacokinetics, reduced off-target side effects, and enhanced delivery efficiency to disease targets (56). There are more kinds of liposomal chemotherapeutic drugs than albumin nanoparticle based chemotherapeutic drugs as various chemotherapeutic drugs could be loaded in liposomes (57). Pegylated liposomal doxorubicin provides comparable efficacy to doxorubicin, with significantly reduced cardiotoxicity, myelosuppression, vomiting and alopecia as first-line therapy for patients with metastatic breast cancer (58). Phase III NAPOLI-1 trial showed that intravenous administration of liposomal irinotecan + 5-FU/LV to gemcitabine-pretreated patients with metastatic pancreatic adenocarcinoma was associated with a prolonged overall survival compared with 5-FU/LV alone (2 months) (59). All these founding demonstrated that nano chemotherapeutic drugs play a vital role in cancer treatment.

## Potential combination of nano-chemotherapy plus immune checkpoint inhibitor

In the combination therapy of chemotherapy plus immune checkpoint inhibitor, only local therapeutic effect of chemotherapy in the tumor region is needed. Jie Mei et al. has confirmed this concept in patients with hecepocellular carcinoma (HCC), which is normally considered chemotherapy insensitive (59). Researchers combined hepatic arterial infusion chemotherapy (HAIC), a local chemotherapy technique, with immune checkpoint inhibitor (PD-1 Inhibitor) and tyrosine kinase inhibitor (Lenvatinib) to treat hepatocellular carcinoma; This treatment scheme showed higher treatment efficiency in hepatocellular carcinoma as the objective response rate (ORR) was 40% with acceptable complications as every patient in the HAIC group finished the treatment with less major adverse events (60). This provides a theoretical basis for the combination of local chemotherapy plus immune checkpoint inhibitor to treat cancers. Based on the above facts, nano chemotherapeutic drugs might have great potential in combination therapy with immune checkpoint inhibitors for cancer patients.

Firstly, concentration of nano chemotherapeutic drugs in tumor region would be higher than traditional chemotherapeutic drugs, improving the therapeutic effect. Compared with the traditional doxorubicin, Doxil (liposomal doxorubicin) showed 4 to 16 times the doxorubicin concentration in the tumor regions of patients using the drug (61). Other studies on the tissue distribution of Doxil using mice models also showed that drug concentration of Doxil in tumor regions was significantly higher than that of free doxorubicin (62). Similarly, animal studies have confirmed that nab-paclitaxel would aggregate in the tumor regions; it also presents a higher bio-availability than its traditional counterpart (63). Jiao-Ren Huang et al. have confirmed that liposomal irinotecan not only increased local drug concentration, but also lasted significantly longer than conventional drugs in the tumor region (64). These evidences showed that nano chemotherapeutic drugs could indeed achieve higher drug concentration and longer existence in tumor area than traditional chemotherapeutic drugs, allowing nano chemotherapy drugs to achieve better clinical therapeutic effect. The combination therapy of liposome irinotecan and 5-fu/lv has improve the overall survival of patients with advanced pancreatic cancer (59); Thus, the treatment schedule is recommended as the second-line treatment for advanced pancreatic cancer. Liposomal doxorubicin has also been recommended by NCCN guidelines for the treatment of ovarian cancer, non Hodgkin’s lymphoma, multiple myeloma, breast cancer, uterine tumor, soft tissue sarcoma and other malignant tumors (65). Nab-paclitaxel has also been approved for the first-line treatment of non-small cell lung cancer and the treatment of advanced pancreatic cancer (53). These evidences further illustrate the advantages of nano chemotherapeutic drugs over traditional chemotherapeutic drugs.

Secondly, nano chemotherapy drugs could reduce systemic toxicity. Almost all the clinical trials of nano chemotherapeutic drugs have proved this point. In a study comparing traditional paclitaxel with nab-paclitaxel, although there was no significant difference in the incidence and degree of side effects between the two drugs, almost every patient in the traditional paclitaxel group received steroid treatment; moreover, patients in the traditional paclitaxel group were more likely to have granulocytopenia above grade 4 (66). A meta-analysis showed that liposomal doxorubicin has less cardio- and other- toxicity than traditional doxorubicin (67). A real-world study found that liposomal doxorubicin could significantly reduce bone marrow suppression, nausea, anorexia and cardiotoxicity caused by traditional chemotherapy (68). These facts not only indicate that patients’ compliance might be improved using nano chemotherapeutic drugs, but also indicate that nano chemotherapeutic drugs could allow more combination treatments for cancer patients. For example, a combination of three drugs is used for advanved pancreatic cancer, including liposomal doxorubicin, PD-1 inhibitor and CXC4 inhibitor; The low toxicity of nano chemotherapeutic drugs greatly enhanced the tolerance of the scheme (69). If the study use traditional doxorubicin instead of liposomal doxorubicin, increasing drop out rate of patients would be observed. Furthermore, due to the decrease of systemic drug concentration, the inhibitory effect of nano chemotherapy drugs on patients’ systemic immune system is also significantly reduced, which might protect the number and the function of T cells, which play an important role in cancer immunotherapy (**Figure 1B**).

In 2019, nab-paclitaxel combined with PD-L1 inhibitor was written into the treatment guidelines for metastatic triple negative breast cancer due to its good therapeutic effect with an ORR rate of 56%, much higher than historical ORR of nab-paclitaxel or PD-L1 inhibitor alone (70). In a phase 2 clinical trial, the authors found the combination of pembrolizumab and liposomal doxorubicin was manageable, without unexpected toxicities, and showed preliminary evidence of clinical benefit in the treatment of platinum resistant ovarian cancer (71). The ORR (26.1%) of combination therapy in this study was higher than that of liposomal doxorubicin (ORR 8.3%) or anti-PD-1/PD-L1 agents (ORR 7.4%) alone in advanced ovarian cancer. Another study focusing on relapsed/refractory classical Hodgkin lymphomaon found that the GVD (gemcitabine, vinorelbine, liposomal doxorubicin)+PD-1 group tended to have a higher CR rate than GVD group (85.2% vs. 65.8%), and had a better event-free survival (the toxicity of the GVD+PD-1 regimen was comparable to the GVD regimen) (72). In addition, in the study of using liposomal irinotecan combined with pembrolizumab (a PD1 monoclonal antibody) and CXCR4 inhibitor to treat pancreatic cancer, the ORR reached 13.2% while the DCR reached 63.2% (60 53). More studies focusing on the combination of nano chemotherapy plus immune checkpoint inhibitor is going on. The ALICE study is planning to compare the therapeutic effect between atezolizumab (PD-L1 inhibitor) plus immunogenic chemotherapy (liposomal doxorubicin + cyclophosphamide) and immunogenic chemotherapy alone on metastatic triple-negative breast cancer (73). Although few clinical studies have been completed so far, these clinical results have already shown that nano chemotherapy drugs combined with immune checkpoint inhibitors might be a potential treatment scheme better than the current traditional chemotherapy plus immune checkpoint inhibitor.

Although clinical evidence is rare, the effectiveness and advantages of therapeutic scheme of nano chemotherapeutic drugs combined with immune checkpoint inhibitors have been confirmed many times *in vivo* experiments. Kuai et al. constructed liposomes loaded with doxorubicin to stimulate the immune system and enhance the efficiency of immunotherapy (74). Results showed that the liposomal doxorubicin ccould trigger a strong CD8+ T cell response without other off-target side effects. When the drug delivery system was further combined with anti-PD-1 antibody, more than 80% of the tumors in mice (both breast and colon cancer models) were completely resolved (74). Moreover, Na Shen et al. constructed P-cis, a kind of cisplatin nanoparticles. Vivo study using tumor mice model showed that P-Cis plus PD1/PD-L1 inhibitors had synergistic and therapeutic advantages compared with traditional cisplatin plus PD1/PD-L1 inhibitors (75).

These *in vivo* studies also confirmed that nano chemotherapeutic drugs could have better local effects, including causing antigen exposure, promoting antigen presentation, and improving the tumor immune micro-environment (76), which would further enhance the anti-tumor function of T cells whose immunosuppressive pathway could be blocked by immune checkpoint inhibitors. With these pre-clinical evidence, it could be expected that more clinical data in the future would be able to confirm the superiority of this scheme over the existing schemes.

Nano chemotherapy drugs could gather in the tumor region and increase the local concentration of drugs. The enhanced permeability and retention (EPR) effect might be the mechanism for the local aggregation (77). There is a hypothesis that the EPR effect is caused by the existence of vascular leakage and damage to the lymphatic system in the tumor. Based on the EPR effect at the tumor site, nano drugs could “passively” accumulate at the site where the vascular permeability increases. In addition, liposomes are not easy to leak into normal tissues with tight endothelial connections so that the side effects of liposomes are significantly reduced compared with free drugs. Active targeting is another way to gather nano chemotherapeutic drugs in the tumor region. In order to achieve the active targeting of cancer sites, a variety of ligands are utilized to exploit any specific antigens expressed by cancer cells, which exhibited increased drug delivery to prostate tumor tissue compared to non-targeting nanoparticles (77). However, there is no active-targeting nano chemotherapeutic drugs applied in clinic currently. Because of their better tumor targeting effect, nano chemotherapeutic drugs with active targeting might be even better than the current nano chemotherapeutic drugs, with more local therapeutic effects and less side effects.

At present, there are few choices of nano chemotherapeutic drugs, which might be due to the limited therapeutic effect of mono-chemotherapy in many advanced tumors, resulting in the slow development of new nano chemotherapeutic drugs. The current treatment scheme of chemotherapy combined with immune checkpoint inhibitors has given new value to nano chemotherapeutic drugs. It does not necessarily lie in the direct anti-tumor effect, but in improving the tumor local immune micro-environment to enhance cancer treatment. Many nano chemotherapeutic drugs use nano vehicles that have already been proved by the FDA, such as liposomes (78). If the treatment scheme could improve the clinical treatment effect for advanced tumors, novel nano chemotherapeutic drugs might soon be developed and put into clinical practice.

## Summary and future perspective

Nano chemotherapy drugs combined with immune checkpoint inhibitors might be a better combination to improve the efficiency of the current scheme of traditional chemotherapy plus immune checkpoint inhibitors in the treatment of tumors. This combination could not only increase the local therapeutic effect of chemotherapy, including increasing antigen presentation and improving the immune micro-environment, thus increasing the therapeutic effect of immune checkpoint inhibitors, but also reduce T cells depletion and systemic toxicity of chemotherapy drugs so that patients would better tolerate this treatment regimen, which would benefit cancer patients ultimately. Current evidence from clinical trials are limited; further validation for its safety and efficiency is needed. Also, novel kinds of nano-chemotherapeutic drugs with better tumor targetability could be expected to improve the therapeutic effect of nano-chemotherapy plus immune checkpoint inhibitors.

## Data availability statement

The original contributions presented in the study are included in the article/supplementary material. Further inquiries can be directed to the corresponding author.

## Author contributions

XQ, WH and JY conceptualized, wrote, and reviewed the manuscript. All authors approved the submitted version.

## Funding

Start up Fund for Talent Researchers of Tsinghua University (No. 10001020507).

## Acknowledgments

We thank Dr. Qian Lu for many fruitful discussions.

## Conflict of interest

The authors declare that the research was conducted in the absence of any commercial or financial relationships that could be construed as a potential conflict of interest.

## Publisher’s note

All claims expressed in this article are solely those of the authors and do not necessarily represent those of their affiliated organizations, or those of the publisher, the editors and the reviewers. Any product that may be evaluated in this article, or claim that may be made by its manufacturer, is not guaranteed or endorsed by the publisher.
